# Beyond proliferation: KLF5 promotes angiogenesis of bladder cancer through directly regulating VEGFA transcription

**DOI:** 10.18632/oncotarget.6101

**Published:** 2015-10-31

**Authors:** Yang Gao, Kaijie Wu, Yule Chen, Jiancheng Zhou, Chong Du, Qi Shi, Shan Xu, Jing Jia, Xiaoshuang Tang, Feng Li, Ke Hui, Dalin He, Peng Guo

**Affiliations:** ^1^ Department of Urology, The First Affiliated Hospital of Xi'an Jiaotong University, Xi'an, Shaanxi, China; ^2^ Oncology Research Lab, Key Laboratory of Environment and Genes Related to Diseases, Ministry of Education, Xi'an, Shaanxi, China

**Keywords:** KLF5, bladder cancer, VEGFA, angiogenesis, endothelial cell

## Abstract

Abundant evidence has demonstrated critical roles of KLF5 in regulating cell proliferation in various cancers, however, its additional roles in other aspects of cancer development remain to be further clarified. In this study, we found that KLF5 was essential for cancer cell-endothelial cell interaction *in vitro* and tumor angiogenesis in nude mice based on lentivirus-mediated KLF5 knockdown bladder cancer cell models. Moreover, KLF5 insufficiency abolished the ability of bladder cancer cells to induce neovascularization in rabbit cornea. Mechanistically, the pro-angiogenic factor VEGFA was identified as a direct downstream target of KLF5, which bound to GC-boxes and CACCC elements of VEGFA promoter and regulated its transcriptional activity. In addition, there was a positive correlation between KLF5 and VEGFA expression in human bladder cancer tissues by immunohistochemistry assay and statistical analysis from TCGA and GEO data. Furthermore, we found that two pivotal pathways in bladder cancer, RTKs/RAS/MAPK and PI3K/Akt, might convey their oncogenic signaling through KLF5-VEGFA axis. Taken together, our results indicate that KLF5 promotes angiogenesis of bladder cancer through directly regulating VEGFA transcription and suggest that KLF5 could be a novel therapeutic target for angiogenesis inhibition in bladder cancer.

## INTRODUCTION

Bladder cancer is one of the most common forms of urological cancer worldwide, with estimated 386,300 new cases and 150,200 deaths per year [1]. In the United States, 74,000 new bladder cancer cases and 16,000 cancer deaths are estimated to occur in 2015 [2]. More than 70% of newly diagnosed bladder cancers are superficial non-muscle invasive bladder cancer (NMIBC), characterized with a high rate of recurrence and certain possibility of progression; while muscle-invasive bladder cancers (MIBC) are extremely aggressive and progress rapidly to become metastatic [3]. Abundant evidence has proved that NMIBC and MIBC have distinct genetic alterations [4], moreover, even in MIBC, distinct molecular subtypes with variable clinical outcomes and responses to conventional chemotherapy have been suggested [5]. All these features reveal the complex characteristics of bladder cancers. Disappointingly, no targeted agent has been approved for treatment of this disease in nearly thirty years, showing an urgent need of research progress to improve diagnosis and treatment of bladder cancer.

Human Kruppel-like factor 5 (KLF5/IKLF/BTEB2), the fifth member of Kruppel-like family, has shown important roles in various physiological and pathological processes including embryonic development, cellular proliferation and differentiation, stress response, cardiovascular remolding and carcinogenesis by regulating the transcription of its target genes [6, 7]. Published data shows that KLF5 is genomically deleted or down-regulated in several types of cancers (i.e., prostate, lung and leukemia) but overexpressed in other types (i.e., breast, esophagus, stomach and colorectal) [6], and these contradictory profiles of KLF5 may be due to its cellular and context-dependent regulation of target gene transcription [8]. It has been reported that exogenous KLF5 expression increased cell cycle transition and up-regulated cyclin D1 in TSU-Pr1 bladder cancer cells [9], and accelerated degradation of KLF5 protein performed by curcumin impaired tumor growth of bladder cancer cells *in vitro* and *in vivo* [10]. On the other hand, during the bladder development of mouse, KLF5 is essential for the formation and terminal differentiation of urothelium [11], and a recently systematic study of human bladder cancer tissues uncovered that *KLF5* was mutated in up to 8% of MIBC, suggesting the importance of this gene in bladder cancer [12]. Given these new findings, the additional roles of KLF5 in other aspects of bladder cancer biology beyond proliferation require to be further investigated.

In this study, using lentivirus-mediated KLF5 knockdown strategy, we found that KLF5 not only revealed its pro-proliferative role in bladder cancer cell lines, but also regulated the interaction between bladder cancer cells and vascular endothelial cells *in vitro* and promoted bladder cancer angiogenesis *in vivo* through directly regulating vascular endothelial growth factor A (VEGFA) transcription. Moreover, KLF5-VEGFA axis could be targeted by inhibiting upstream oncogenic signaling pathways such as receptor tyrosine kinases (RTKs) and phosphoinositide 3-kinase (PI3K).

## RESULTS

### KLF5 knockdown inhibited cell proliferation in a subset of bladder cancer cells *in vitro* and in 5637 xenografts *in vivo*

**Figure 1:**
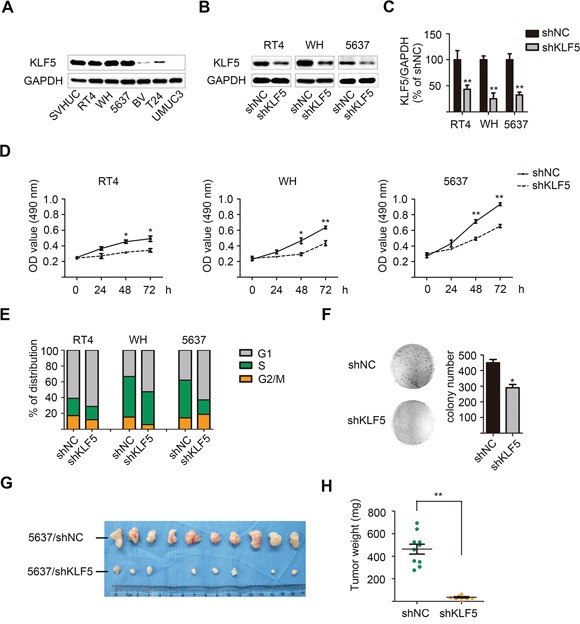
Lentivirus-mediated KLF5 knockdown suppresses the proliferation of bladder cancer cells *in vitro* and *in vivo* **A.** Expression of KLF5 in selected bladder normal/cancer cells as detected by western blot. GAPDH was used as internal control. Effects **B.** and quantification **C.** of non-specific (NC) or KLF5 shRNA-bearing lentivirus in knockdown of KLF5 in three bladder cancer cell lines. These results represent three independent experiments. **D.** Differences of growth rate between shNC and shKLF5 clones determined by MTT assay. **E.** Cell cycle distribution of these clones detected by flow cytometry. **F.** Colony-formation abilities of 5637/shNC and 5637/shKLF5 clones. **G.** Subcutaneous xenografts of 5637/shNC and 5637/shKLF5 clones harvested 3 weeks after injection. **H.** Tumor weights between the two groups. The values were shown as the mean ± SD. *p < 0.05, **p < 0.01.

In non-tumor urothelial cell line SVHUC and six bladder cancer cell lines, RT4, WH and 5637 cells were found to express relatively high level of KLF5 protein and mRNA (Figure 1A, and Supplementary Figure S1). To further clarify roles of KLF5 in bladder cancer, we applied lentivirus-delivered specific shRNA to knockdown KLF5 in these three cell lines (Figure 1B–1C). Consistent with our previous studies, KLF5 still played a critical role in the regulation of cell proliferation in bladder cancer cells. Compared with the shNC cells, RT4, WH and 5637 cells with KLF5 knockdown showed impaired cellular proliferation by MTT assays (Figure 1D). In addition, flow cytometry assay results showed significant cell cycle arrest in G1 phase after KLF5 knockdown accordingly (Figure 1E).

Furthermore, the colony-forming abilities *in vitro* and subcutaneous tumorigenesis potential *in vivo* of 5637/shKLF5 cells were significantly reduced compared with 5637/shNC cells (Figure 1, 1F–1G). In the nude mice xenograft model, 3 weeks after implantation, the tumor weight in 5637/shKLF5 group (37.13 ± 6.072 mg, *n* = 8) dramatically decreased to be less than 10% of the 5637/shNC group (463.5 ± 44.35 mg, *n* = 10; Figure 1H). However, this was not consistent with our *in vitro* observation showing 30% decrease of cell growth in 5637/shKLF5 cells. Therefore, we suppose that other effects (such as cell-cell interaction in tumor microenvironment) may play a critical role in the modulation of *in vivo* tumor growth by KLF5 beyond regulating autonomous cancer cell proliferation.

### KLF5 regulated interactions between bladder cancer cells and HUVECs

**Figure 2:**
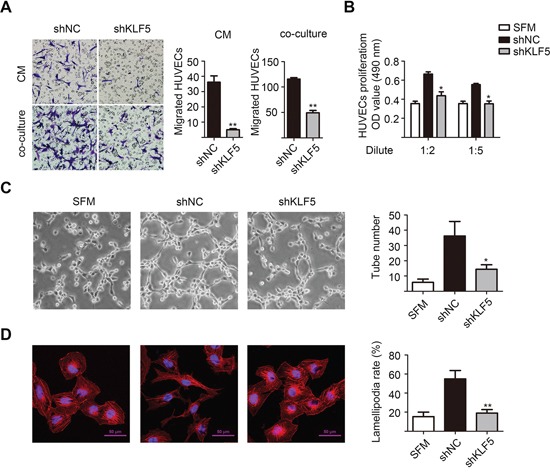
KLF5 is essential for the interaction between bladder cancer cells and HUVECs **A.** KLF5 knockdown decreased the recruitment of HUVECs. Conditioned medium (CM) collected from the 5637/shNC and 5637/shKLF5 cells or these cancer cells cultured in the bottom of 24-well plate were used to recruit HUVECs seeding on the upper side of the transwell insets within 16 hours. Migrated cells in 6 random fields (200 ×) per well were counted. **B.** KLF5 knockdown reduced the proliferation of HUVECs. HUVECs treated with serum free medium (SFM) or diluted CMs for 48 hours were detected by MTT assay. **C.** KLF5 knockdown suppressed tube formation of HUVECs. HUVECs diluted in SFM or CMs were added into matrigel-coated wells and incubated for 4 hours. Representative photographs of tube-like structures were taken and tube numbers in the whole field were counted (right). **D.** KLF5 knockdown decreased the lamellipodia formation of HUVEC cells. Serum-starved HUVECs were stimulated with SFM or CMs for 15 minutes, then fixed and stained with rhodamine-phalloidin for F-actin and DAPI for nucleus. Cells with lamellipodia structure were counted and lamellipodia formation rates were calculated. Scale bars = 50 μm. These data were representative of three independent experiments. Bars, SD; *p < 0.05, **p < 0.01 compared with shNC group.

Indeed, all 5637/shKLF5 xenograft tumors were pale and almost no blood vessels were shown on the surface of tumor capsule, indicating a deficient angiogenesis after KLF5 knockdown. To reveal the potential roles of KLF5 in angiogenesis, we collected the conditioned mediums (CMs) of these shNC and shKLF5 subclones. In an *in vitro* endothelial recruitment assay, human umbilical vein endothelial cells (HUVECs) were seeded onto transwell inserts and different CMs were added into the lower chambers as attraction. After incubation for 16 hours, CMs from shKLF5 subclones of RT4, WH and 5637 showed reduced abilities to recruit HUVECs compared with their controls (Figure 2A, upper panel and Supplementary Figure S2, S2A–S2B). In a co-cultured system, 5637/shKLF5 cells also recruited less HUVECs than 5637/shNC cells (Figure 2A, lower panel). Moreover, we found that CMs from 5637/shNC cells but not 5637/shKLF5 cells maintained the growth of HUVECs in serum-deprived condition (Figure 2B) and the formation of tube-like structures in matrigel (Figure 2C). Furthermore, we stimulated serum-starved HUVECs with CMs from 5637/shNC or shKLF5 cells and stained with rhodamine-phalloidin for F-actin to detect a potential cytoskeleton change under confocal microscopy. Indeed, HUVECs treated with CMs from 5637/shNC cells formed more lamellipodia structures than those treated with CMs from shKLF5 cells (Figure 2D). Therefore, these results suggest that loss of KLF5 may reshape the paracrine characteristics of bladder cancer cells and affect the interactions between bladder cancer cells and HUVECs.

### VEGFA as a target of KLF5 mediated its function in angiogenesis

**Figure 3:**
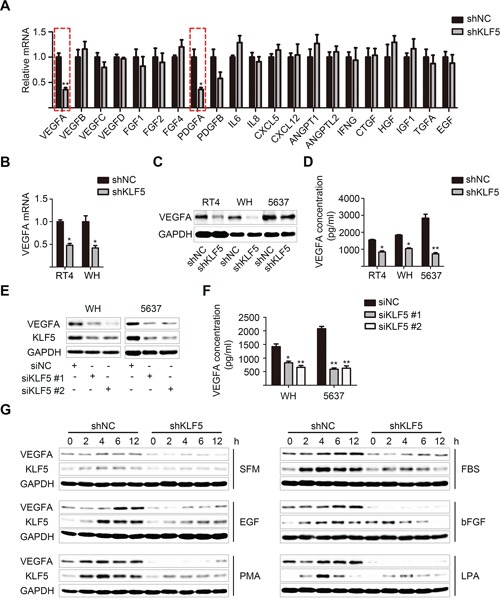
KLF5 regulates VEGFA expression in bladder cancer cells **A.** Real-time PCR was used to profile differently expressed angiogenic cytokines between 5637/shNC and 5637/shKLF5 cells. **B.** KLF5 knockdown reduced the expression of VEGFA in RT4 and WH bladder cancers. **C.** VEGFA protein level in these KLF5-knockdown bladder cancer cell models was detected by western blot analysis. **D.** Concentrations of secreted VEGFA protein in the CMs were determined by ELISA. **E.** WH and 5637 cells were transfected with siRNAs specifically targeting KLF5 (si KLF5 # 1,2) or non-specific control (si NC). Expression of KLF5 and VEGFA were analyzed 48 hours after transfection. **F.** Secreted VEGFA in CMs of the siRNAs transfected cells was detected by ELISA assay. **G.** Serum starved 5637/shNC and 5637/shKLF5 cells were treated with SFM, FBS (5%), EGF (50 ng/ml), PMA (50 nM) and LPA (10 μM) for indicated periods of time. Then KLF5 and VEGFA levels were measured by western blot assay. The values were shown as the mean ± SD. *p < 0.05, **p < 0.01.

Because KLF5 is a DNA-binding protein and usually promotes transcription of multiple target genes, we assume that one or more angiogenic factors may be involved in the interactions between bladder cancer cells and HUVECs after KLF5 knockdown. Therefore, we performed real-time qPCR assay to profile differently expressed angiogenic cytokines between 5637/shNC and 5637/shKLF5 cells. Among the 21 candidates, the change of VEGFA and platelet-derived growth factor alpha polypeptide (PDGFA) showed statistical differences (Figure 3A). Because VEGFA plays predominant roles in tumor angiogenesis, we decide to focus our investigation on the regulation of VEGFA by KLF5 and its roles in bladder cancer angiogenesis. Consistently, real-time qPCR and western blot assays also showed a significantly reduced VEGFA mRNA and protein expression after KLF5 knockdown in all three cell lines (Figure 3, 3B–3C). In addition, ELISA data further showed the lower VEGFA concentrations detected in the CMs from shKLF5 cells compared with those from shNC cells (Figure 3D), which might differently affect HUVECs. Furthermore, in WH and 5637 cells, two differently-designed KLF5-specific siRNAs effectively inhibited both KLF5 and VEGFA expression, decreased VEGFA secretion in the medium and abolished HUVECs recruitment compared with the negative control group (Figure 3, 3E–3F and Supplementary Figure S3). Previous reports have shown that KLF5 could be upregulated by fetal bovine serum (FBS), growth factors such as epidermal growth factor (EGF), basic fibroblast growth factor (bFGF), phorbol-12-myristate-13-acetate (PMA) and lysophosphatidic acid (LPA) [7]. We observed that these stimuli time-dependently elevated both KLF5 and VEGFA expression in 5637/shNC cells, but not in 5637/shKLF5 cells (Figure 3G), indicating that KLF5 might mediate the induction of VEGFA by upstream signals.

**Figure 4:**
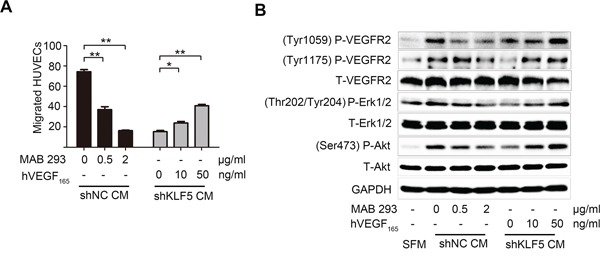
VEGFA mediates the angiogenic roles of KLF5 in bladder cancer **A.** VEGFA neutralized antibody (MAB293) was added into CMs from 5637/shNC cells, while recombinant human VEGF_165_ was added into CMs from 5637/shKLF5 cells. These CMs containing different levels of VEGFA were used to recruit HUVECs. **B.** HUVECs were serum-starved overnight, stimulated with SFM or CMs for 10 minutes before proteins harvest immediately. Phosphorylation of VEGFR2, Erk1/2 and Akt were evaluated by western blot. These data were representative of three independent experiments; *p < 0.05, **p < 0.01.

To further verify the importance of KLF5-regulated VEGFA in bladder cancer angiogenesis, we modified VEGFA concentration in the CMs using VEGFA neutralizing antibody MAB293 or recombinant human VEGF_165_. Significantly, CMs from 5637/shNC cells incubated with VEGFA neutralizing antibody recruited less HUVECs, while VEGF_165_ addition into CMs from 5637/shKLF5 cells restored the angiogenic abilities (Figure 4A). Consistently, we also observed the different phosphorylation of VEGFR2, Akt and Erk1/2 signaling pathways in HUVECs treated with CMs from 5637/shNC or 5637/shKLF5 cells, which could be modulated by changing VEGFA concentration with MAB293 or VEGF_165_ (Figure 4B). Taken together, these data demonstrate that KLF5 regulated bladder cancer angiogenesis through promoting VEGFA expression.

### KLF5 initiated VEGFA transcription through directly binding to GC/CACCC boxes in VEGFA promoter

**Figure 5:**
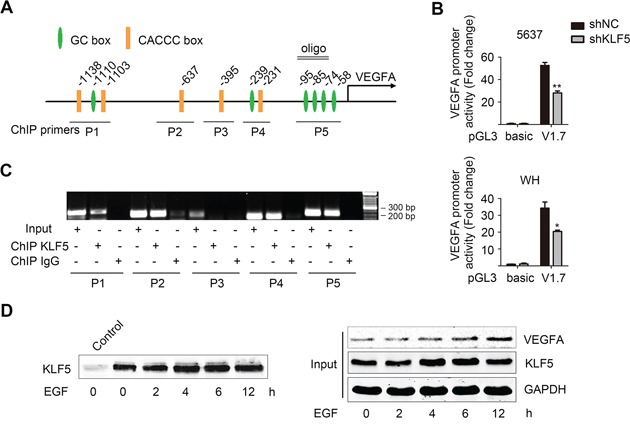
KLF5 directly binds to VEGFA promoter and regulates its transcription activity **A.** Schematic illustration of human VEGFA promoter, putative KLF5 binding sites (GC box and CACCC box), oligo design (for oligo pulldown assay) and primers used in ChIP assay. **B.** pRL-TK and pGL3-basic or pGL3-V1.7 (harboring a 1.7 kb human VEGFA promoter fragment) plasmids were co-transfected into 5637/shNC and 5637/shKLF5 cells (upper), and also WH/shNC and WH/shKLF5 cells (lower). Reporter activities were analyzed by dual luciferase activity assay. *p < 0.05, **p < 0.01, compared with shNC group. **C.** Evaluation of KLF5 binding to the VEGFA promoter region by ChIP assay. DNA was prepared from normally cultured 5637 cells and then antibodies against KLF5 were used to pulldown the corresponding protein-bound DNA. **D.** Oligonucleotides pull-down assays were performed in 5637 cells treated with 50 ng/ml EGF for indicated time. KLF5 was detected by western blot assay. Non oligonucleotide group was used as control.

KLF5 has been shown to bind to the GC boxes and CACCC boxes in promoters of its target genes, therefore we searched and identified 11 putative KLF5 binding elements within 1700bp ahead of the transcription start site (Figure 5A). Then we cloned this DNA fragment and inserted it into the pGL3-basic luciferase reporter plasmid. Using pRL-TK as internal control, we performed dual luciferase activity assay in 5637 and WH subclones. The pGL3-V1.7 plasmids in shKLF5 cells showed a significantly decreased activity compared with those in control cells (Figure 5B), which was consistent with the reduced VEGFA mRNA expression in these KLF5 knockdown cells. Five paired primers covering separated regions in VEGFA promoter were used in ChIP assay. As shown in Figure 5C, 4 of 5 regions containing GC boxes or CACCC boxes maintained higher binding affinity with KLF5. To verify whether KLF5 directly bound to region of −102/−63, which is proximal to the transcription start site of *VEGFA* promoter, biotinylated oligonucleotides with the same sequences were synthesized and used to pull-down KLF5 proteins in 5637 cells after EGF treatment. Along with the EGF treatment, more KLF5 bound to VEGFA promoter, which might lead to an increased VEGFA expression (Figure 5D).

### KLF5 regulated VEGFA expression and tumor angiogenesis *in vivo*

**Figure 6:**
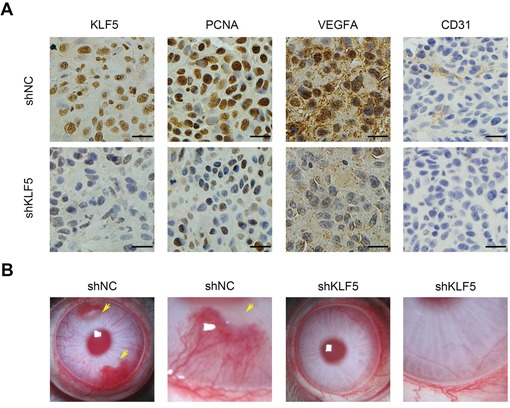
KLF5 is required for tumor angiogenesis *in vivo* **A.** Representative photograph (400 ×) of immunohistochemistry (IHC) staining of KLF5, PCNA, VEGFA and CD31 expression in paraffin fixed tumor sections from 5637/shNC and 5637/shKLF5 xenografts. Bars = 20 μm. **B.** 5637/shNC cells but not 5637/shKLF5 cells could induce angiogenesis in rabbit cornea (*n* = 4). Neonatal vessels invaded the cornea, connected the tumor (yellow arrow) and the limbal vascular plexus.

*In vivo*, we stained KLF5 and VEGFA in those 5637 xenograft tissues as described in Figure 1G by immunohistochemistry (IHC) assays. The reduced KLF5 expression accompanied by less VEGFA staining in 5637/shKLF5 tumors was observed. Consistently, staining of proliferating cell nuclear antigen (PCNA) and CD31 (endothelial cell marker) showed similar results (Figure 6A). Furthermore, we performed a more specific and sensitive rabbit cornea assay to verify the pro-angiogenic role of KLF5. Three weeks after transplanted into cornea, 5637/shNC cells induced neovascular response and formed visible tumors, but 5637/shKLF5 lost these abilities (Figure 6B). These data suggested that KLF5 was required for 5637 bladder cancer angiogenesis in animal models.

### KLF5 and VEGFA co-expressed in human bladder cancer tissues

**Figure 7:**
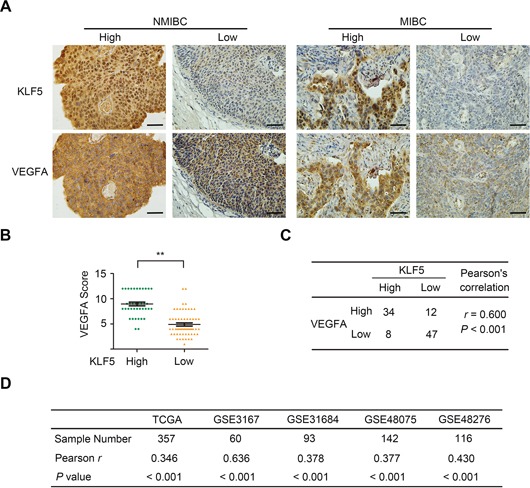
Co-expression of KLF5 and VEGFA in human bladder cancer tissues **A.** Representative photographs (400 ×) showed IHC staining for KLF5 and VEGFA in non-muscle invasive bladder cancer (NMIBC, *N* = 52) and muscle invasive bladder cancer (MIBC, *N* = 49). Bars = 50 μm. **B.** Comparing stain scores of VEGFA between KLF5 high expression (score > 6) and low expression (score ≤ 6) groups. Bars, SEM; **p < 0.01. **C.** Statistic analysis of KLF5 and VEGFA protein co-expression. **D.** Microarray data downloaded from TCGA and four GEO datasets was analyzed for the correlation between KLF5 and VEGFA mRNA.

We also investigated KLF5 and VEGFA expression in 101 human transitional bladder cancer tissues using IHC assays. According to the clinical stage, tissues were separated into two groups: NMIBC (pTa and pT1) and MIBC (pT2-4). As shown in Figure 7A, KLF5 was located on both cellular cytoplasm and nucleus while VEGFA was maintained on cellular membrane and cytoplasm. In the KLF5 highly expressed tissues (IHC score > 6), the expression of VEGFA proteins were significantly higher (8.952 ± 0.3686, *n* = 42) compared with those in KLF5 low expressed tissues (4.898 ± 0.3186, *n* = 59, Figure 7B). Statistical analysis suggested that KLF5 and VEGFA were highly co-expressed in these tissues (*r* = 0.600, *p* < 0.01; Figure 7C). Furthermore, microarray data profiling of human bladder cancer tissues downloaded from TCGA and GEO database was applied to examine the co-expression of KLF5 and VEGFA. As shown in Figure 7D, analysis of five cohorts (768 patients in total) in which both KLF5 and VEGFA mRNA data was available supported the positive correlation between KLF5 and VEGFA. Importantly, KLF5 as well as VEGFA expression was positively correlated with neovascularization (as evaluated by CD31 staining) in bladder cancer tissues (Supplementary Figure S4A–S4C). Thus, clinical evidence above supports that KLF5 might play roles in angiogenesis of human bladder cancer through regulating VEGFA expression.

### PMA/LPA/RTKs-KLF5-VEGFA signaling as therapeutic targets in bladder cancer

**Figure 8:**
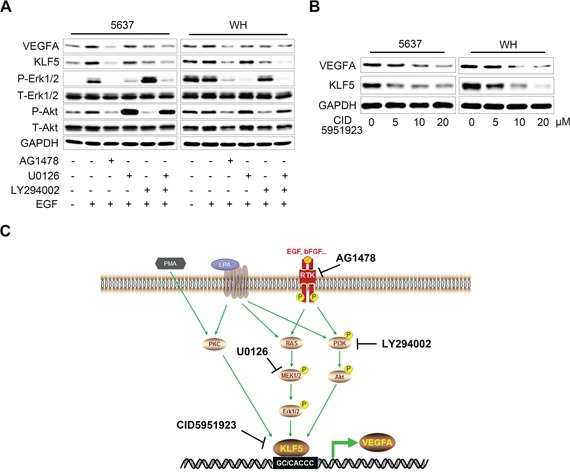
Therapeutic potential of targeting KLF5 in bladder cancer cells **A.** Serum-starved 5637 and WH cells were pre-treated with inhibitors of EGFR (AG1478, 5 μM), MEK1/2 (U0126, 5 μM) or PI3K (LY294002, 5 μM) before EGF (50 ng/ml) treatment for 12 hours. Then KLF5, VEGFA expression and phosphorylation status of Erk1/2 and Akt were evaluated by western blot. **B.** Impact of a KLF5 specific inhibitor (CID5951923) on the KLF5 and VEGFA protein expression as measured by western blot in 5637 and WH cells. **C.** Schematic model of PMA/LPA/RTKs-KLF5-VEGFA signaling and novel therapeutic targets in bladder cancer.

As shown above, growth factors (EGF and bFGF) and stimuli (PMA and LPA) that activated downstream oncogenic signaling (such as PKC, MEK/Erk and PI3K/Akt) could elevate the expression of KLF5, then increased VEGFA transcriptional efficiency and promoted bladder cancer angiogenesis. Thus, we also investigated the potential therapeutic value of targeting these molecules in bladder cancer. As shown in Figure 8A, in both 5637 and WH cells, EGFR inhibitor AG1478, MEK1/2 inhibitor U0126 or PI3K inhibitor LY294002 all showed the inhibition on EGF-induced KLF5 and VEGFA expression. Importantly, a small-molecule compound CID5951923 which specifically inhibited KLF5 transcription [13], was also applied and showed the suppression on both KLF5 and VEGFA expression in the two cells in a dose-dependent manner (Figure 8B). Therefore, KLF5-VEGFA axis could be regulated by RTKs/RAS/MAPK and PI3K/Akt pathways, which might provide a novel therapeutic target in bladder cancer (Figure 8C).

## DISCUSSION

In the present study, we found that pro-proliferative factor KLF5 was not only required for bladder cancer cell growth, but also essential for the interaction between bladder cancer cells and endothelial cells *in vitro* and tumor angiogenesis in the xenografts in nude mice and in rabbit cornea. Mechanistically, KLF5 exerts these functions as a novel pro-angiogenic factor in bladder cancer by directly regulating the transcription of VEGFA, which is the most prominent factor among the angiogenic cytokines [14–16]. In the present study, we demonstrated that several GC boxes and CACCC elements in promoter of VEGFA served as binding sites for KLF5, and knockdown of KLF5 significantly impaired VEGFA promoter transcription activity. This findings were similar with its regulation on FGF-BP in breast cancer cells [17]. Moreover, at both mRNA level (TCGA and GEO data) and protein level (IHC staining), KLF5 and VEGFA were found to be co-expressed in human bladder cancer tissues, further supporting that KLF5 could be an essential regulator of VEGFA in bladder cancer. It has been reported that conditional deletion of *KLF5* in the lung of mouse inhibited the maturation of lung during the saccular stage of development and simultaneously decreased VEGFA mRNA level, but the underlying mechanism had not been detailed [18]. Our data here not only elucidate the mechanism how KLF5 activates the transcription of VEGFA, but also provide evidence of the correlation between expression of KLF5 and VEGFA in human bladder cancer cell lines and tissues. On the other hand, the protein levels of KLF5 and VEGFA are correlated with the expression of CD31, a marker of neovascularization, in human bladder cancer tissues and tumor xenografts in nude mice. Taken together, our results suggest that KLF5 may play an important role in the angiogenesis of bladder cancer through regulating VEGFA.

Two independent studies of high throughput genomic sequencing of human bladder cancer tissues uncovered that frequent alterations of RTKs/RAS/MAPK and PI3K/Akt/mTOR signaling pathways were critical features of bladder cancer [12, 19], suggesting that both pathways could be important targets in the treatment of bladder cancer [20]. Interestingly, KLF5 is a downstream factor of both pathways. Its transcriptional activity was regulated by MAPK effector Egr-1, which could be activated by RTK ligands or mutant HRAS [7, 8], on the other hand, both LY294002 and wortmannin, inhibitors of PI3K, could efficiently down-regulate KLF5 promoter transcriptional activity in DLD-1 colorectal cancer cells [21]. We reported here that in bladder cancer cells, KLF5-VEGFA axis was activated by growth factor EGF and bFGF, PKC agonist PMA and mitogenic lipid LPA, but inhibited by inhibitors of EGFR (AG1478), MEK1/2 (U0126) or PI3K (LY294002). Our results suggest that VEGFA is stimulated by various extracellular signals through different pathways, such as RTKs/RAS/MAPK and PI3K/Akt/mTOR, while they converge on the same downstream gene KLF5 and KLF5 subsequently mediates the activation of VEGFA transcription by these signals (Figure 8C). Thus, inhibition of KLF5 could avoid the bypass of different pathways and it could be a better strategy to suppress VEGFA in bladder cancer. Furthermore, CID 5951923, a specific KLF5 transcriptional inhibitor identified by cell-based ultrahigh-throughput screening [13], abolished KLF5 and VEGFA expression in 5637 and WH bladder cancer cells, suggesting potential application of KLF5 inhibitor in bladder cancer treatment.

Seems to be contradictory, a latest study in prostate cancer reported that KLF5 inhibited angiogenesis in PTEN-deficient prostate cancer by attenuating Akt activation and subsequent HIF1-maintained VEGFA expression [22]. Actually, these results were not exclusive with our finding, because: (1) KLF5 has been proved to be a transcription factor that switches roles from transcriptional activator to repressor, or from cell growth promoter to inhibitor, or from oncogene to tumor suppressor, depending on the cellular and genetic context [6–8]. (2) In prostate cancer, KLF5 is frequently deleted and down-regulated [23]. Overexpression of KLF5 suppressed the growth of prostate cancer cell line DU145 and 22RV1 [23]. However, our studies in bladder cancer all support KLF5 as a pro-proliferative factor [7, 9]. KLF5 might function differently and organ-specifically in prostate cancer and bladder cancer. (3) Our previous studies uncovered that TGFβ1 signaling increased the acetylation of KLF5, reversed its regulation on p15 and MYC, switched its roles from pro-proliferative to anti-proliferative in epithelial cells [24, 25]. And modification of KLF5 acetylation status also converted its roles in prostate cancer [26]. Thus whether KLF5 acetylation levels are different between prostate cancer and bladder cancer and between *PTEN* deleting statuses remain to be clarified.

In cardiovascular system, roles of KLF5 in angiogenesis have also been suggested because of its direct regulation of PDGFA in cardiac fibroblasts and smooth muscle cells [9, 27–29]. In our study, knockdown KLF5 suppressed PDGFA expression in 5637 cells. We could not rule out the potential effects of PDGFA in KLF5-regulated angiogenesis, because VEGFA neutralizing antibody could not absolutely abolish the recruit of HUVECs by 5637/shNC cells and recombinant human VEGF_165_ could not completely restore the angiogenic abilities in 5637/shKLF5 cells. Recently, PDGFA has been proved to play important roles in breast cancer [30], but whether PDGFA serve as important factor in bladder cancer development has not been evidenced. Thus further study may be needed to uncover the roles of PDGFA in KLF5-regulated angiogenesis in bladder cancer.

Although KLF5 has similar effect on angiogenesis in non-muscle invasive bladder cancer (NMIBC) and muscle-invasive bladder cancers (MIBC), NMIBC and MIBC are different in many aspects including genetic mutations. Thus, the context-dependent transcription factor KLF5 may exert different functions other than tumor angiogenesis in these two types of bladder cancers, which is needed to be further clarified in the future. On the other hand, be aware of the limitation of cell line based research models, spontaneous bladder cancer mice model and tissue specific inducible *KLF5* deletion mouse model as reported previously in mouse intestine could be better models for us to reveal the function of KLF5 in bladder cancer development in our future studies [31].

In conclusion, we reported that knockdown of proliferative factor KLF5 not only suppressed the growth of bladder cancer cells but also impaired bladder cancer cell-endothelial cell interaction. KLF5-insufficient 5637 cells showed decreased tumorigenic ability in nude mice and failed to induce a neovescular response in rabbit cornea. One of the underlying mechanism was that KLF5 directly bound to the promoter region of VEGFA and regulated its transcription activity. Moreover, suppression of KLF5-VEGFA axis by the inhibitors of upstream oncogenic signaling pathways (such as RTKs and PI3K) and inhibitor of KLF5 suggested KLF5 as a potential therapeutic target in bladder cancer.

## MATERIALS AND METHODS

### Cell lines and reagents

Human umbilical vein endothelial cell line HUVEC, SV40 immortalized human urothelial cell line SVHUC, bladder papillary tumor cell line RT4 and bladder cancer cell lines WH and BV were kindly provided by Dr. Jer-Tsong Hsieh (University of Texas Southwestern Medical Center, Dallas, TX, USA). Bladder cancer cell lines 5637, T24 and UMUC3 were generously provided by Dr. Leland W. K. Chung (Cedars-Sinai Medical Center, Los Angeles, CA, USA). The 5637 cells were cultured in RPMI 1640 and other cells were cultured in DMEM with 10% fetal bovine serum at 37°C with 5% CO_2_.

Recombinant human EGF and bFGF were purchased from R&D Systems Inc. (Minneapolis, MN, USA). Phorbol-12-myristate-13-acetate (PMA) and lysophosphatidic acid (LPA) were obtained from Sigma–Aldrich (St. Louis, MO, USA). All the reagents were reconstituted and stored following the protocol.

### Patients and tissue samples

Paraffin-embedded tissues from 101 bladder cancer patients after TURBT or cystectomy in Department of Urology, The First Affiliated Hospital of Xi'an Jiaotong University were used for immunohistochemistry (IHC) study of KLF5, VEGFA and CD31 expression. Tissue procurement protocol has been approved by the Institutional Review Board and appropriate informed consent was obtained from all patients. Clinicopathologic characteristics of patients were listed in Table 1.

**Table 1:** Clinicopathologic characteristics of patients

Characteristic	Number (%)
Total	101 (100%)
Age	
< 60 years	37 (37%)
≥ 60 years	64 (63%)
Gender	
Male	78 (77%)
Female	23 (23%)
Tumor stage	
pTa, pT1	52 (51%)
pT2	24 (24%)
pT3	19 (19%)
pT4	6 (6%)

### Bioinformatics

Bladder cancer microarray data from four GEO DataSets (GSE3167, GSE31684, GSE48075 and GSE48276) were downloaded from GEO website (http://www.ncbi.nlm.nih.gov/geo/) and RNA sequencing data of 357 bladder cancer samples was downloaded from TCGA Data portal (https://tcga-data.nci.nih.gov/tcga/tcgaHome2.jsp) at Jan 25, 2015. All the data were normalized to Z score before statistical analysis [32].

### Plasmids, siRNA, transfection, lentivirus preparation and infection

PLKO.1 lentiviral vectors encoding short hairpin RNA (shRNA) targeting non-specific control (NC) or human KLF5 (sh710: 5′-GGTTACCTTACAGTATCAACA-3′) were constructed by GenPharma (Shanghai, China). The siRNAs targeting KLF5 and non-specific control (NC) were purchased from RiboBio (RiboBio Co., Ltd., Guangzhou, China). The specific sequences of the *KLF5* gene for siRNA to target were: 5′-GCTCCAGAGGTGAACAATA-3′ (#1) and 5′-CAACCTGTCAGATACAATA-3′ (#2). To generate lentivirus, PAX2, VSV-G and the plasmids described above were co-transfected into 293T cells using the Lipofectamine 2000 reagent (Invitrogen, CA, USA) according to the manufacturer's protocol. After 72 hours, the supernatants were harvested and used to infect RT4, WH and 5637 cells in the presence of 8 μg/ml polybrene. The stable KLF5 knockdown cell clones were selected and maintained in 2–3 μg/ml puromycin.

### MTT assay

Growth rates of cells were measured by 3-(4, 5-dimethylthiazol-2-yl)-2, 5-diphenyltetrazolium bromide (MTT) assay as described previously [10].

### Colony formation assay

5637/shNC and 5637/shKLF5 cells were seeded onto 6 well plates at a density of 1000 cells per well. After 72 hours of culture, cells were washed with PBS, fixed with 4% paraformaldehyde, and subsequently stained with 0.1% crystal violet solutions. All the visible cell colonies formed in a well were counted. The experiments were performed in triplicate.

### Cell cycle analysis

Cells with 60–80% confluence were trypsinized, washed with cold PBS, resuspended with cold 70% ethanol then stored at −20°C for at least 24 hours. Before subjected to flow cytometry analysis, removed ethanol and washed pellet cells with cold PBS twice, resuspended cells in PBS with 0.5 μg/ml RNAse A and 50 μg/ml propidium Iodide and incubated at room temperature in the dark room for 30 minutes.

### Conditioned medium collection, ELISA assay and HUVEC stimulation

Bladder cancer cells were seeded at 5 × 10^5^ cells per 60 mm culture dish. After adhesion, cells were washed with serum free medium (SFM) for three times, feed with 5 ml SFM and cultured for 24 hours. Then the supernatants were collected and centrifuged to remove cell debris. Conditioned mediums (CMs) were stored in −80°C before use. RayBio^®^ Human VEGF ELISA Kit (RayBiotech Inc, Norcross, GA, USA) was used for quantitative measurement of human VEGF in CMs. Modification of VEGF concentration in the CMs was performed by addition of neutralizing VEGF antibody (MAB293, R&D Systems Inc., Minneapolis, MN, USA) or addition of recombinant Human VEGF_165_ (R&D Systems Inc., Minneapolis, MN, USA). Serum-starved HUVECs were treated with CMs above for indicated time, then were fixed with 4% paraformaldehyde for morphological studies or lysed for western blot assay.

### HUVEC migration assay

Transwell migration assays were performed in 8-μm-pore transwell inserts (Millipore, Bedford, MA, USA). 300 μl HUVECs suspended in SFM at 3 × 10^5^ cells/ml were seeded into upper chamber and 1 ml of medium containing different conditioned mediums were added to the lower chamber as a chemoattractant. After incubation for 16 hours, the upper surface of the insert was wiped with a cotton swab and cells that migrated to the lower surface were fixed by 4% paraformaldehyde and stained with crystal violet. Cell numbers were counted in 6 random fields (200 ×) per well.

### Tube formation assay

HUVECs (1 × 10^5^/well) suspended in SFM or SFM diluted (1:1) conditioned medium were seeded into matrigel-coated wells of a 24-well plate. Four to six hours later, photographs were taken. Only perfectly continuous tubes between 2 branching points were considered as a tube.

### Immunofluorescence microscopy

The immunofluorescence staining of F-actin in HUVECs was performed according to the manufacturer's protocol as we previously described [33]. Micrographs were obtained with Nikon A1R confocal microscope (Tokyo, Japan).

### Real-time quantitive PCR (qPCR) assay

Cells were harvested with Trizol reagent (Life Technologies, Rockville, MD, USA) to extract total RNA, which was reverse-transcribed to cDNA using PrimeScript™ RT reagent kit (Takara, Dalian, China). Then the cDNA was studied using CFX96 real-time PCR system (Bio-Rad, Hercules, CA) using SYBR Green PCR Master Mix (Takara, Dalian, China) to determine the transcriptional expression of specific genes (primer sequences shown in Supplementary Table S1). GAPDH was used for normalization. Relative gene expression was calculated by the 2^−ΔΔCt^ method.

### Western blot analysis

Cells were washed once with cold PBS and lysed in RIPA buffer (50 mM Tris pH 8.0, 150 mM NaCl, 0.1% SDS, 1% NP-40, and 0.5% sodium deoxycholate) containing protease inhibitors. Approximate 30 μg of protein was separated with 8–12% SDS–PAGE gel and blotted onto nitrocellulose membranes. Then membranes were blocked with 5% skim milk at room temperature for 1 hour and then incubated with primary antibodies against GAPDH (Shanghai Kangchen), KLF5 [34], VEGFA (Abcam), total and phosphorylated VEGFR2, Erk1/2, and Akt (Cell signaling Technology) at 4°C overnight, followed by TBST wash and 1 hour incubation with horseradish peroxidase-conjugated secondary antibodies at room temperature. Protein bands were visualized by a Molecular Imager ChemiDoc XRS System (Bio-Rad Laboratories, Hercules, CA, USA).

### Dual luciferase activity assay

VEGFA promoter report plasmids pGL3-V1.7 were generated by inserting a 1718 bp promoter region (−1618 to +100) of VEGFA into pGL3-basic plasmids. The primers used in amplified PCR were: forward, 5′-ATTCCCATTCTCAGTCCATG-3′; reverse, 5′-CTGACCGGTCCACCTAACCG-3′. To perform promoter luciferase assay, ERE-TK-Luc, pGL3-basic and pGL3-V1.7 were co-transfected into KLF5-knockdown subclones of 5637 and WH cells using X-tremeGENE HP DNA transfection reagent (Roche, Mannheim, Germany). Luciferase assay was carried out using the Dual Luciferase Assay kit (Promega, Madison, WI, USA) following the manufacturer's instructions. Three wells of cells were used for each data point.

### Chromatin immunoprecipitation assay

ChIP assay was performed in normal cultured 5637 cells using SimpleChIP^®^ Enzymatic Chromatin IP Kit (Magnetic Beads) from Cell Signal Technology (Danvers, MA, USA) following the manufacturer's protocol. Antibody against KLF5 [34] or normal rabbit IgG were used to precipitate protein/DNA complex. Precipitated DNA was analyzed by PCR with region-specific primers (Supplementary Table S2).

### Oligonucleotides pull-down assay

Oligonucleotides for the VEGFA promoter (−102 to −63), with biotin added to their 5′-end, were synthesized by GENEWIZ (Suzhou, China). The oligonucleotide sequence was: biotin-5′-CCTGTCCC CG CCCCCCGGGGCGGGCCGGGGGCGGGGTCC-3′. 5637 cells were serum-starved overnight and then treated with 50 ng/ml EGF for the indicated time before lysed. Procedures for pull down DNA-bound proteins were detailed in our previously study [35]. Finally, the KLF5 protein was detected by western blot.

### Xenograft tumor model and rabbit cornea assay

For tumorigenesis assay in nude mice, 2 × 10^6^ cells were injected subcutaneously into both sides of the flank region. Five mice were used for each cell clone. Xenograft tumors were harvested, weighted and fixed with 4% paraformaldehyde after 3 weeks. For rabbit assay in New Zealand white rabbits, a micropocket (1.5 × 3 mm) was surgically produced in the 6 and 12 o'clock points of the right eye. 1.5 × 10^5^ 5637/shNC or 5637/shKLF5 cells were implanted into the micropockets (four rabbits per group). After 3 weeks, angiogenic responses in corneas were evaluated. Animal care and protocols were in accordance with the guidelines of the Institutional Animal Care and Use Committee of Xi'an Jiaotong University.

### Immunohistochemistry

Tumor sections of bladder cancer patients or nude mice xenografts were studied by immunohistochemistry (IHC) assay using EnVisionTM System (DAKO, Carpinteria, CA, USA). Primary antibodies used in IHC were KLF5 (Abcam, ab24331, 1:200), PCNA (Santa Cruz, sc-7907, 1:300), VEGFA (Abcam, ab46154, 1:150) and CD31 (Epitomics, 2530-1, 1:100). Staining signals were photographed using an Olympus BX51 Microscope (Olympus, Tokyo, Japan). Average intensity score of the positive cells (0-none, 1-weak, 2-intermediate, and 3-strong) and percentage score of stained cells (1–0% to 25%, 2–25% to 50%, 3–50% to 75%, and 4–75% to 100%) were multiplied to get the total staining score, ranging from 0 to 12. Neovessel number in a bladder cancer tissue was counted following the previously described method [36].

### Statistical analysis

GraphPad Prism version 6.0 software (GraphPad, San Diego, CA, USA) was used to analyze differences between two groups (student's *t*-test), calculate Pearson's correlation and perform linear regression analysis. A *p* value less than 0.05 was considered to be significant.

## SUPPLEMENTARY FIGURES AND TABLES


