# Supplementary material for: Beyond proliferation: KLF5 promotes angiogenesis of bladder cancer through directly regulating VEGFA transcription
Source: Oncotarget. 2015 Oct 31;6(41):43791–805. doi: 10.18632/oncotarget.6101 (PMC4791267; doi:10.18632/oncotarget.6101)
Supplement: Supplementary file 1 [file oncotarget-06-43791-s001.pdf]

SUPPLEMENTARY FIGURES AND TABLES

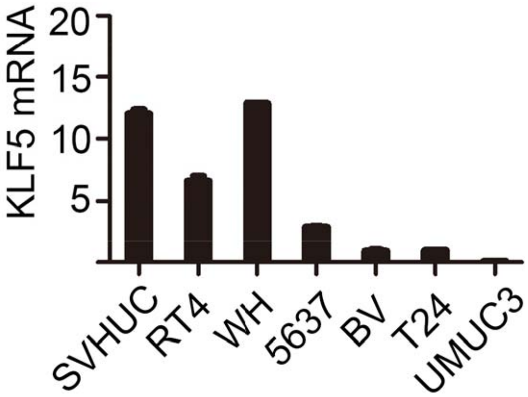

Supplementary Figure S1: Expression of KLF5 mRNA in selected bladder normal/cancer cells. Total RNA was reverse-transcribed to cDNA. Then KLF5 expression levels were studied using real-time PCR. GAPDH was used as internal control. Results were presented as the mean ± SD from three independent experiments.

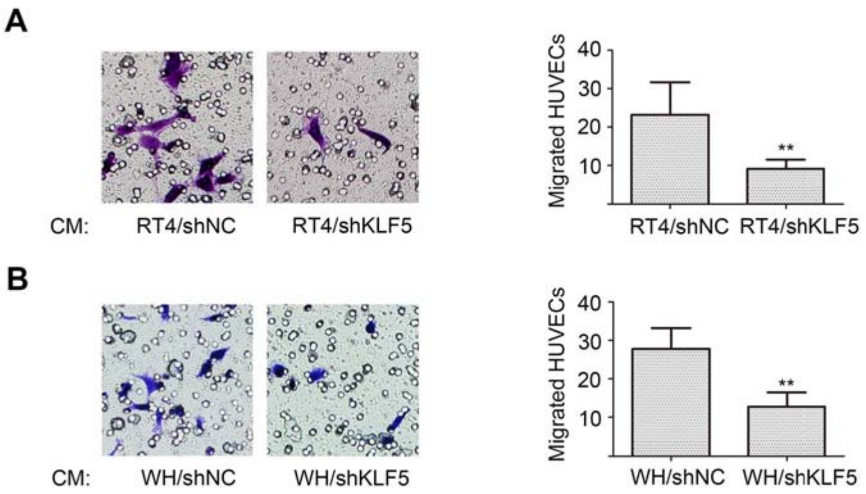

Supplementary Figure S2: KLF5 knockdown by lentivirus in RT4 and WH cells impaired their angiogenic abilities. A. Conditioned mediums (CMs) collected from the RT4/shNC and RT4/shKLF5 cells were used to recruit HUVECs seeding in the upper side of the trans-well insets within 16 hours. Migrated cells in 6 random fields (200×) per well were counted. **B.** The same assay in WH/shNC and WH/shKLF5 clones. These results represent three independent experiments. The values are the mean ±SD. $^{**}p < 0.01$ compared with shNC group.

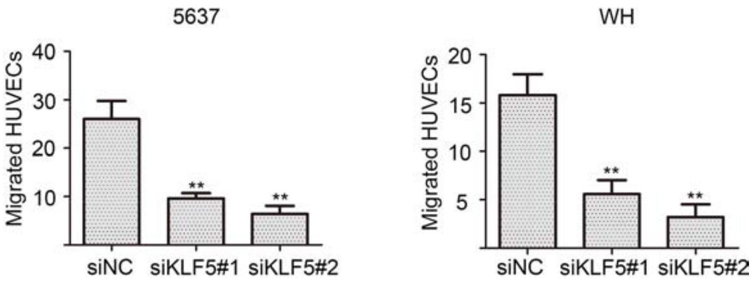

Supplementary Figure S3: KLF5 knockdown by siRNAs in 5637 and WH cells abolished HUVECs recruitment by CMs. 5637 and WH cells were transfected with siRNAs specifically targeting KLF5 (si KLF5 # 1,2) or non-specific control (si NC). Conditioned mediums (CMs) collected 72 hours after transfection were used to recruit HUVECs. These results represent three independent experiments. The values are the mean ±SD. $^{**}p < 0.01$ compared with shNC group.

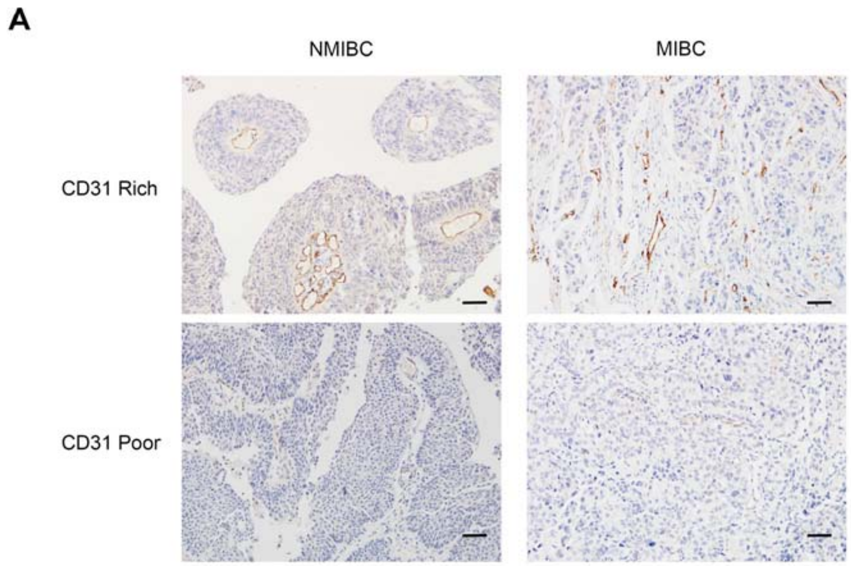

B

		KLF5 High	KLF5 Low	Pearson's correlation
CD31	Rich	27	24	$r = 0.233$
	Poor	15	35	$P < 0.05$

C

		VEGFA High	VEGFA Low	Pearson's correlation
CD31	Rich	33	18	$r = 0.389$
	Poor	13	37	$P < 0.01$

Supplementary Figure S4: Correlation of KLF5 and VEGFA expression with CD31 positive neovessel number in human bladder cancer tissues. A. Representative photographs (200 ×) showed IHC staining for CD31 in NMIBC and MIBC. Bars = 50 μm. According to the average vessel number per "neovascular hot spots", tissues were divided into "CD31 Rich" (vessel number > = 6) and "CD31 Poor" (vessel number < 6) groups. **B, C.** Statistic analysis the correlation between KLF5 (B) and VEGFA (C) expression with neovascularization.

Supplementary Table S1: Primer sequences used in real-time qPCR

Gene	Forward primer	Reverse primer
GAPDH	ATGGGGAAGGTGAAGGTCGG	GACGGTGCCATGGAATTTGC
KLF5	CAGAGGACCTGGTCCAGACAAG	GAGGCCAGTTCTCAGGTGAGTG
VEGFA	GAGCCTTGCCTTGCTGCTCTA	CACCAGGGTCTCGATTGGATG
VEGFB	GAGATGTCCCTGGAAGAACACA	GAGTGGGATGGGTGATGTCAG
VEGFC	GAGGAGCAGTTACGGTCTGTG	TCCTTTCCTTAGCTGACACTTGT
VEGFD	ATGGACCAGTGAAGCGATCAT	GTTCCTCCAAACTAGAAGCAGC
FGF1	CTCCCGAAGGATTAAACGACG	GTCAGTGCTGCCTGAATGCT
FGF2	AGAAGAGCGACCCTCACATCA	CGGTTAGCACACACTCCTTTG
FGF4	CTCGCCCTTCTTCACCGATG	GTAGGACTCGTAGGCGTTGTA
PDGFA	GCAAGACCAGGACGGTCATTT	GGCACTTGACACTGCTCGT
PDGFB	CTCGATCCGCTCCTTTGATGA	CGTTGGTGCGGTCTATGAG
IL6	CCTGAACCTTCCAAAGATGGC	TTCACCAGGCAAGTCTCCTCA
IL8	ACTGAGAGTGATTGAGAGTGGAC	AACCCTCTGCACCCAGTTTTC
CXCL5	AGCTGCGTTGCGTTTGTTTAC	TGGCGAACACTTGCAGATTAC
CXCL12	ATTCTCAACACTCCAAACTGTGC	ACTTTAGCTTCGGGTCAATGC
ANGPT1	AGCGCCGAAGTCCAGAAAAC	TACTCTCACGACAGTTGCCAT
ANGPTL2	TCCTGCACGAGATCATCCG	GGTGCTGGTACTTGTGCTCC
IFNG	TCGGTAACTGACTTGAATGTCCA	TCGCTTCCCTGTTTTAGCTGC
CTGF	AAAAGTGCATCCGTACTCCCA	CCGTCGGTACATACTCCACAG
HGF	GCTATCGGGGTAAAGACCTACA	CGTAGCGTACCTCTGGATTGC
IGF1	GCTCTTCAGTTCGTGTGTGGA	GCCTCCTTAGATCACAGCTCC
TGFA	AGGTCCGAAAACACTGTGAGT	AGCAAGCGGTTCTTCCCTTC
EGF	TGATTGAAATGGCCAATCTGGA	GCATAGCCCAATCTGAGAACCAC

Supplementary Table S2: Primer sequences used in ChIP assay

	Forward primer	Reverse primer	Range#
P1	CTTGGGCTGATAGAAGCCTT	ACAGAGGCCCTTGTTCCTGC	−1217 to −1022; 195bp
P2	CAGCCCTGGGCTCTCTGTAC	CTCCATTCACCCAGCTTCCC	−731 to −533; 198bp
P3	TCTTCGAGAGTGAGGACGTG	CTAGGAATATTGAAGGGGGC	−524 to −342; 182bp
P4	CCTAGCAAAGAGGGAACGGC	CTCGACCCCCACCAAGGTTC	−347 to −193; 154bp
P5	GTCGAGCTTCCCCTTCATTG	CCTCCCCGCTACCAGCCGAC	−199 to −1; 198bp

#transcript start site = 0.
